# Gut microbiome–micronutrient interaction: The key to controlling the bioavailability of minerals and vitamins?

**DOI:** 10.1002/biof.1835

**Published:** 2022-03-16

**Authors:** Monica Barone, Federica D'Amico, Patrizia Brigidi, Silvia Turroni

**Affiliations:** ^1^ Microbiomics Unit, Department of Medical and Surgical Sciences University of Bologna Bologna Italy; ^2^ Unit of Microbiome Science and Biotechnology, Department of Pharmacy and Biotechnology University of Bologna Bologna Italy

**Keywords:** absorption, biosynthesis, deficiency, gut microbiome, micronutrients, minerals, vitamins

## Abstract

Micronutrients, namely, vitamins and minerals, are necessary for the proper functioning of the human body, and their deficiencies can have dramatic short‐ and long‐term health consequences. Among the underlying causes, certainly a reduced dietary intake and/or poor absorption in the gastrointestinal tract play a key role in decreasing their bioavailability. Recent evidence from clinical and in vivo studies suggests an increasingly important contribution from the gut microbiome. Commensal microorganisms can in fact regulate the levels of micronutrients, both by intervening in the biosynthetic processes and by modulating their absorption. This short narrative review addresses the pivotal role of the gut microbiome in influencing the bioavailability of vitamins (such as A, B, C, D, E, and K) and minerals (calcium, iron, zinc, magnesium, and phosphorous), as well as the impact of these micronutrients on microbiome composition and functionality. Personalized microbiome‐based intervention strategies could therefore constitute an innovative tool to counteract micronutrient deficiencies by modulating the gut microbiome toward an eubiotic configuration capable of satisfying the needs of our organism, while promoting general health.

**Abbreviations**
DAP: 1,3‐diaminopropane
SCFAs: short‐chain fatty acids

## 1 INTRODUCTION

Micronutrients include organic and inorganic elements and compounds, such as minerals and vitamins, which are essential for the maintenance of host health and are not used for energy balance. Such micronutrients are commonly found in foods and dietary supplements, and are crucial for the regulation of biosynthetic cellular reactions, for example, those involved in immune and energy functions,^1^ as well as in biological processes such as growth, bone health, and fluid balance.^2^ Inadequate levels of micronutrients, resulting from reduced intake and/or poor absorption, are known to lead to specific micronutrient deficiency diseases, which represent a major global health concern.^3^, ^4^ Micronutrient deficiencies can also aggravate infections and noncommunicable chronic diseases, such as osteoporosis, hypothyroidism, cardiovascular disease and cancer, with a potentially dramatic impact on quality of life, morbidity and mortality.^3^, ^5^, ^6^ For example, in children, vitamin D deficiency leads to poor physical and mental development, and contributes to the onset of inflammatory diseases and allergies.^7^, ^8^ Vitamin D deficiency may contribute to unbalanced immune responses even in adults, resulting in a higher incidence and progression of autoimmune diseases.^7^, ^9^ Among the most widespread intervention strategies to improve the micronutrient status thus reducing the disease burden, large‐scale fortification and public health programs must certainly be mentioned. Nonetheless, a growing body of evidence has highlighted some concerns about the negative consequences of supplementing certain micronutrients.^4^, ^10^ Oral iron supplementation has been linked to an increased incidence of constipation, gastric irritation, nausea, and metallic taste.^11^ For infants and young children, the consumption of iron‐fortified foods has been associated with decreased growth,^12^ impaired cognitive development,^13^, ^14^ and increased diarrhea probably due to altered to increased levels of intestinal pathogenic bacteria (e.g., *Escherichia coli*).^15^, ^16^ In addition, excessive vitamin A intake has been linked to increased bone fracture in both men and women.^17^, ^18^ Furthermore, despite supplementation, some deficiencies, such as those of iron, vitamin A, and zinc, have been shown to persist in certain individuals, underlining the involvement of other determinants of bioavailability.^4^

The gut microbiome, that is, the community of trillions of microorganisms that inhabit our gastrointestinal tract, is known to interact with dietary compounds in a bidirectional way, being impacted in its compositional and functional structure, as well as influencing their metabolism and absorption, therefore their bioavailability.^19^, ^20^, ^21^ As regards specifically micronutrients, these can modulate the diversity and composition of the gut microbiome, leading to beneficial or vice versa detrimental outcomes for the host health.^22^, ^23^ On the other hand, commensal bacteria in the human gut are able to influence nutrient absorption^24^ and to synthesize essential vitamins (e.g., vitamin K and biotin, among others),^25^ thus having a potentially considerable impact on the micronutrient status.

In this short narrative review, our aim is to provide up‐to‐date evidence on the two‐way interaction between gut microorganisms and micronutrients. In particular, we summarize the available in vivo and clinical studies dealing with the gut microbiome role in influencing micronutrient bioavailability and the modulation of the microbiome by micronutrients. We will also provide some glimpses on the modulation of the gut microbiome to improve the host micronutrient status. An increased understanding of the underlying mechanisms may indeed pave the way for the development of microbiome‐based intervention strategies to reduce micronutrient imbalances and related diseases, thereby limiting unwanted supplementation and improving overall human health.

## 2 GUT MICROBIOME–MICRONUTRIENT INTERACTIONS

Microbes utilize micronutrients for their growth and biological functioning. It is therefore not surprising that the intake of micronutrients can influence the compositional and functional structure of the gut microbiome. For example, dietary supplementation with vitamin B, C, D, and E largely contributes to the microbiome composition, by favoring the expansion and colonization of the intestinal mucosa by beneficial genera such as *Bifidobacterium*, *Lactobacillus*, and *Roseburia*.^26^, ^27^, ^28^, ^29^ Minerals such as calcium, iron, zinc, magnesium, and phosphorous can also shape the human gut microbiome.^21^, ^22^ In particular, high calcium intake has been associated with a higher proportion of *Clostridium* cluster XVIII in men,^30^ iron supplementation could induce a depletion of *Bifidobacterium* and an increase in *Lactobacillus* levels in children,^31^ and phosphorous supplementation leads to an increase in microbial diversity and levels of short‐chain fatty acids (SCFAs) in stool.^30^ On the other hand, it has been shown that various members that make up the intestinal microbial ecosystem can influence the bioavailability of micronutrients, by controlling their absorption, particularly phosphorous and calcium,^32^ and synthesizing vitamins, such as vitamin K and the water‐soluble fraction of B vitamins.^33^

**FIGURE 1:**
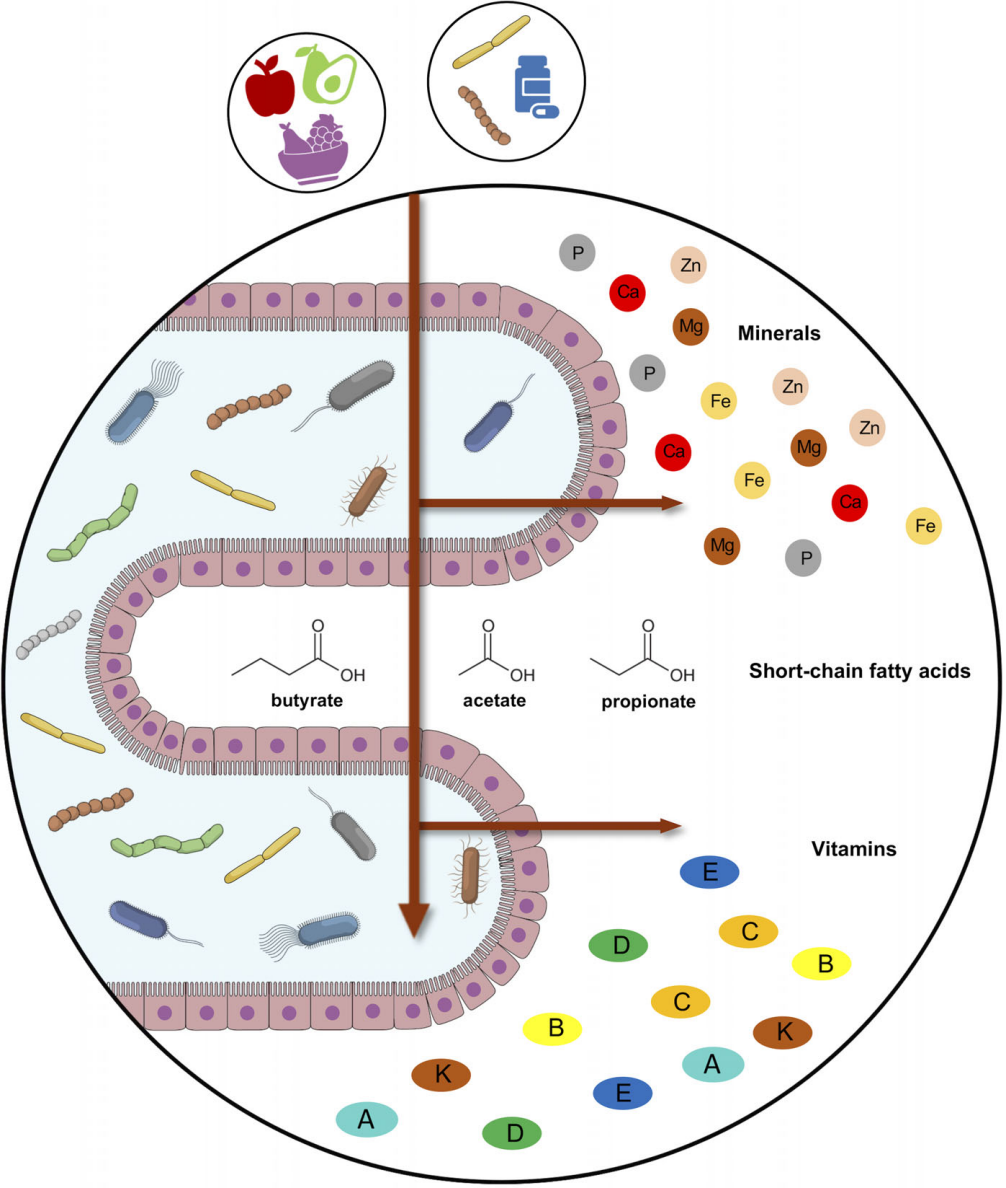
Micronutrient interchange between the gut microbiome and the host. Micronutrients and the gut microbiome interact along a bidirectional axis. Dietary micronutrients can in fact affect the composition and functionality of the microbiome, and the latter can influence the bioavailability of micronutrients, for example, by synthesizing vitamins and controlling the absorption rate. In particular, the absorption seems to be favored by the generation of short‐chain fatty acids, end‐products of the microbial fermentation of polysaccharides. Microbiome‐based intervention strategies, such as prebiotics, probiotics, and postbiotics, could therefore be the key to improving deficiency conditions, thereby limiting potentially harmful supplementation practices

Below we will discuss the most recent in vivo and clinical studies on the bidirectional interaction between gut commensals and micronutrients, that is, minerals (calcium, iron, zinc, magnesium, and phosphorous) and vitamins (A, B, C, D, E, and K). Figure 1 shows the summary of microbiome–micronutrient interactions in the gut.

### 2.1 Minerals

The human gut microbiome comprises several bacteria that possess the ability to affect the host mineral status, both through the synthesis of a wide range of enzymes involved in the release of minerals from dietary sources, and by directly influencing the absorption rate at the gastrointestinal level.^21^

Among bacterial enzymes, phytases are capable of releasing bioavailable forms of inorganic minerals, such as calcium, magnesium, iron, and phosphorous, following the hydrolysis of phytic acid present in many plant‐based foods consumed with the diet.^34^ Furthermore, a correlation was observed between the relative abundance of SCFA‐producing members of the gut microbiome and the calcium absorption rate in in vivo and clinical studies, as well as with bone mineral density in animal models.^35^, ^36^ SCFAs (microbial metabolites resulting from polysaccharide fermentation) actually lower the pH of the gastrointestinal tract, facilitating the solubility of calcium and consequently enhancing its transepithelial transport, as demonstrated in vitro with rat cecum and colon preparations.^37^ In addition to calcium, Wang et al. recently observed gut microbiome‐dependent amelioration in bone phosphorous content, following oral administration of the probiotic *Enterococcus faecium* to broilers.^38^ Again, the effect was likely attributable to the production of SCFAs, which increase mineral solubilization by lowering the pH and could interact directly with the skeleton (increasing bone mass and preventing bone loss),^39^ as well as an increased phytase activity of the microbiome. Calcium and phosphate form amorphous calcium complexes in the small intestine, allowing both bile and fatty acids to bind,^40^, ^41^ and affecting the composition of the gut microbiome.^41^, ^42^ Among the major effects, an increase in butyrate and acetate producers such as *Clostridium* spp. and consequently fecal levels of SCFAs have been observed in a recent clinical trial following oral administration of phosphorous and calcium.^43^ However, it is still unclear whether the above associations result from calcium, phosphorous or a combination of the two minerals.^30^

Clinical trials focusing on the administration of iron‐containing micronutrient powders to infants at higher risk of diarrhea and respiratory tract infections, have shown that iron supplementation negatively modulates microbiome composition, resulting in decreased relative abundance of health‐associated taxa (i.e., bifidobacteria and lactobacilli), increased proportions of pathogens, and higher levels of plasma intestinal fatty acid‐binding protein, which acts as a biomarker of enterocyte damage.^15^, ^16^, ^44^, ^45^ Iron is indeed known to promote the growth and virulence of various enteropathogens, for example, by increasing the production of toxic metabolites or by inducing the biosynthesis of flagella, thus facilitating colonization and invasion of the epithelia, and contributing to an overall proinflammatory state.^46^ On the other hand, similarly to what has been discussed above, iron absorption in the intestine is influenced by microbial metabolites, more precisely promoted by SCFAs,^47^, ^48^ while hindered by 1,3‐diaminopropane and the antimicrobial reuterin (3‐hydroxypropionaldehyde) produced by *Lactobacillus reuteri*.^49^

Low zinc levels represent a significant risk factor for poor health outcomes, with women and children being the most vulnerable targets.^4^, ^50^, ^51^ In vivo studies have shown that zinc deficiency alters the gut microbiome composition, with a decrease in biodiversity, an increase in inflammatory markers and an impairment of the functional potential involved in gut–brain signaling.^52^, ^53^ As demonstrated in an in vitro digestion model, the gut microbiome could negatively affect zinc bioaccessibility by reducing the dissolution of food‐derived zinc in the colon.^54^ It is therefore not surprising that clinical trials on probiotic supplementation for the treatment of zinc deficiency have shown conflicting results. In particular, the first available clinical trial showed no significant changes in mineral bioavailability following probiotic supplementation with *Lactobacillus casei* and *L. reuteri* in pediatric cohorts.^55^ In contrast, the second clinical trial achieved a significant increase in zinc levels after *Lactobacillus plantarum* and zinc supplementation, only when the two supplements were administered simultaneously.^56^ Despite the small size of the enrolled pediatric cohort, a recent clinical trial by Ballini et al.^57^ showed that supplementation with a synbiotic containing the probiotic species *L. plantarum*, *Lactobacillus acidophilus*, *Bifidobacterium infantis*, and *Bifidobacterium lactis*, in combination with the prebiotic fructooligosaccharide, could be useful for raising blood zinc levels.^57^

Finally, several studies in animal models have pointed out associations between magnesium bioavailability and gut microbiome composition.^58^, ^59^, ^60^ The few available studies in humans suggest that gut commensals and probiotic strains belonging to *Lactobacillus* spp. are effective in increasing the bioavailability of magnesium after cheese and vegetable milk consumption.^61^, ^62^, ^63^ On the other hand, magnesium has been shown to exert a positive impact on the gut microbiome composition, as well as on the metabolism of vitamins B1 and D, in patients with metabolic syndrome, type 2 diabetes and obesity.^64^

### 2.2 Vitamins

Some bacterial genera normally adapted to the human gastrointestinal tract niche, including, *Bifidobacterium*, *Bacteroides*, and *Enterococcus*, are well‐known to biosynthesize vitamin K and those of group B, more precisely the water‐soluble forms of the latter.^23^, ^65^, ^66^ Furthermore, as reported for minerals, the gut microbiome can affect vitamin absorption rates as well as be affected by dietary supplementation, as will be detailed in this section.

With specific regard to B vitamins, in a systematic review, Magnusdottir et al. evaluated in silico the potential of 256 common human gut commensals to produce biotin, cobalamin, folate, niacin, pantothenate, pyridoxine, riboflavin, and thiamine.^67^ According to them, human gut microorganisms produce 40 to 65% of these vitamins, supporting host‐microbiome coevolution. Nevertheless, in order to be fully available to the host, the de novo biosynthesis of vitamins must necessarily take place upstream of their absorption site. In this perspective, since vitamin B12 can only be absorbed at the level of the ileum, cobalamin‐producing microorganisms in the large intestine are unlikely to contribute to its actual bioavailability.^68^ On the other hand, dietary supplementation of B vitamins has been associated with changes in the diversity and composition of the gut microbiome. For example, vitamin B3 supplementation has been correlated with the expansion of Bacteroidetes, along with improved biomarkers of metabolic inflammation and insulin sensitivity.^69^ In addition, clinical trials focusing on the oral administration of vitamin B2 have shown the enrichment of butyrate producers such as *Faecalibacterium* and *Roseburia* in healthy individuals,^70^ as well as the depletion of *Enterobacteriaceae* in patients with inflammatory bowel disease.^71^ However, in a second clinical trial, Pham et al. did not replicate the increase in *Faecalibacterium*, but still observed an increase in diversity,^72^ reinforcing the hypothesis of a modulatory potential of B vitamins on the human gut microbiome.

Using a model of intestinal enteroids treated with lipopolysaccharide, Subramanian et al. have shown that the gut microbiome may also negatively affect the absorption rate of vitamin C, by downregulating the transcription of sodium‐dependent vitamin C transporters.^73^ On the other hand, vitamin C supplementation has been shown to significantly increase the microbial ecosystem biodiversity, along with the relative abundance of *Collinsella* and fecal levels of SCFAs, particularly butyrate and propionate, when compared to the placebo group.^72^ An in vitro association has also been observed with several health‐promoting taxa (i.e., *Roseburia*, *Faecalibacterium*, *Akkermansia*, and *Bifidobacterium*), but the trend has not yet been confirmed in humans.^72^

Although fat‐soluble vitamins are not naturally produced by gut commensals, significant associations have been found between this subgroup of vitamins and the gut microbiome. For instance, in a large‐scale clinical study focusing on the host micronutrient status from the microbiome point of view, serum vitamin D levels were positively correlated with several members of the Firmicutes phylum, such as *Ruminococcus*, *Coprococcus*, *Mogibacterium*, and *Blautia*.^72^, ^74^ The modulatory effects of vitamin D are likely due to the control of the expression of antimicrobial peptides and the protective effects on the gut mucosa, that is, the maintenance of barrier integrity and epithelial healing.^75^, ^76^, ^77^ In addition, a previous clinical study by Jones et al. highlighted a relationship between vitamin D and *L. reuteri*, probably mediated by the metabolism of bile acids, with important therapeutic repercussions given the widespread use of this microorganism as a probiotic.^78^ Finally, the dietary intake of vitamin E has been positively associated with SCFA production, as well as the relative abundance of *Akkermansia* and other health‐promoting taxa such as *Lactobacillus*, *Bifidobacterium*, and *Faecalibacterium*.^79^ On the other hand, other in vivo studies have found a negative association between the gut microbiome and the bioavailability of vitamin E. For instance, Ran et al. reported an increase in the absorption rate of vitamin E after antibiotic treatment in a mouse model, suggesting vitamin degradation by intestinal commensals.^80^

## 3 MICROBIOME‐BASED INTERVENTION STRATEGIES TO IMPROVE THE HOST MICRONUTRIENT STATUS

The aforementioned literature on the bidirectional gut microbiome–micronutrient axis strongly suggests that personalized microbiome‐based interventions could be a promising tool for improving host micronutrient status. Tailored interventions aimed at restoring/maintaining a eubiotic microbiome profile, capable of producing vitamins and SCFAs while being low in lipopolysaccharides, could be instrumental to ensure adequate biosynthesis and absorption of micronutrients, for optimal bioavailability for the host. For instance, prebiotic galactooligosaccharides mitigated the adverse effects of iron supplementation on the infant gut,^81^ while the administration of *L. reuteri* in combination with the laxative magnesium oxide ameliorated chronic constipation in children without causing imbalances in the microbiome.^82^

Further clinical studies that integrate multiomics approaches are needed to identify the bacterial species involved and assess their actual contribution to the micronutrient balance, hence their role in preventing and/or ameliorating any deficiencies. By elucidating the mechanisms underlying the relationship between our microbial counterpart and micronutrient bioavailability, postbiotic strategies could also be developed to broaden the range of precision personalized interventions aimed at tackling micronutrient deficiencies and related disorders.

## 4 CONCLUDING REMARKS

The gut microbiome can variously influence the bioavailability of micronutrients, as well as be influenced by micronutrient supplementation, with implications for health even in the long term. Although several mechanisms have been advanced, a thorough characterization of the microbiome–micronutrient bidirectional axis is of utmost importance, as it can guide the design of microbiome‐based precision intervention strategies, aimed at improving micronutrient status and overall health.

## CONFLICTS OF INTEREST

The authors declare no conflicts of interest.

## Data Availability

Data sharing not applicable ‐ no new data generated, or the article describes entirely theoretical research.
